# Circadian clock and lipid metabolism disorders: a potential therapeutic strategy for cancer

**DOI:** 10.3389/fendo.2023.1292011

**Published:** 2023-12-22

**Authors:** Mengsi Liu, Zhen Zhang, Yating Chen, Ting Feng, Qing Zhou, Xuefei Tian

**Affiliations:** ^1^ School of Integrated Chinese and Western Medicine, Hunan University of Chinese Medicine, Changsha, Hunan, China; ^2^ Hunan Key Laboratory of Traditional Chinese Medicine Prescription and Syndromes Translational Medicine, Hunan University of Chinese Medicine, Changsha, China; ^3^ Hunan Province University Key Laboratory of Oncology of Traditional Chinese Medicine, Changsha, China; ^4^ Key Laboratory of Traditional Chinese Medicine for Mechanism of Tumor Prevention and Treatment, Hunan University of Chinese Medicine, Changsha, China; ^5^ Department of Oncology, Affiliated Hospital of Hunan Academy of Traditional Chinese Medicine, Changsha, China; ^6^ Department of Andrology, The First Affiliated Hospital of Hunan University of Chinese Medicine, Changsha, China

**Keywords:** circadian clock, lipid metabolism, cancer, fatty acid synthetic, SREBP

## Abstract

Recent research has emphasized the interaction between the circadian clock and lipid metabolism, particularly in relation to tumors. This review aims to explore how the circadian clock regulates lipid metabolism and its impact on carcinogenesis. Specifically, targeting key enzymes involved in fatty acid synthesis (SREBP, ACLY, ACC, FASN, and SCD) has been identified as a potential strategy for cancer therapy. By disrupting these enzymes, it may be possible to inhibit tumor growth by interfering with lipid metabolism. Transcription factors, like SREBP play a significant role in regulating fatty acid synthesis which is influenced by circadian clock genes such as BMAL1, REV-ERB and DEC. This suggests a strong connection between fatty acid synthesis and the circadian clock. Therefore, successful combination therapy should target fatty acid synthesis in addition to considering the timing and duration of drug use. Ultimately, personalized chronotherapy can enhance drug efficacy in cancer treatment and achieve treatment goals

## 1 Introduction

Cancer is a major global public health issue due to its high incidence and the metabolic reprogramming, immune evasion, proliferative signaling, growth suppression avoidance, cell death resistance, replication immortality, angiogenesis induction, invasion and metastasis activation it entails (1). Tumor cells undergo lipid metabolic reprogramming through *de novo* lipogenesis involving key transcription factors such as sterol regulatory element binding protein (SREBP), ATP citrate lyase (ACLY), acetyl-CoA carboxylase (ACC), fatty acid synthase (FASN) and stearoyl-CoA desaturase 1 (SCD1). Unhealthy lifestyle habits like shift work disrupt circadian rhythm which can contribute to the development of diabetes and cancer. Shift work is classified as a potential human carcinogen due to its disruption of circadian rhythms that regulate crucial biological processes leading to abnormal cell proliferation, gene mutation, and resistance to apoptosis (1–3). Research has also demonstrated that circadian clock genes and lipid metabolism play a role in cancer development by regulating signaling pathways and metabolites.

In this article, we have provided a comprehensive analysis of lipid metabolism and clock genes, with focus on SREBP as key factor. We have summarized the impact of transcription factors involved in *de novo* lipogenesis on lipid metabolism, as well as their mechanism of intersection with circadian clock genes. Furthermore, we have examined the interaction relationship between transcription factors responsible for *de novo* fatty acid synthesis, clock genes and cancer. Lastly, we have reviewed the advancements in utilizing circadian clock and lipid metabolism in cancer treatment and discussed their potential application in clinical therapies.

## 2 Circadian clock and lipid metabolism

### 2.1 The molecular mechanism of circadian clock genes

**Figure 1:**
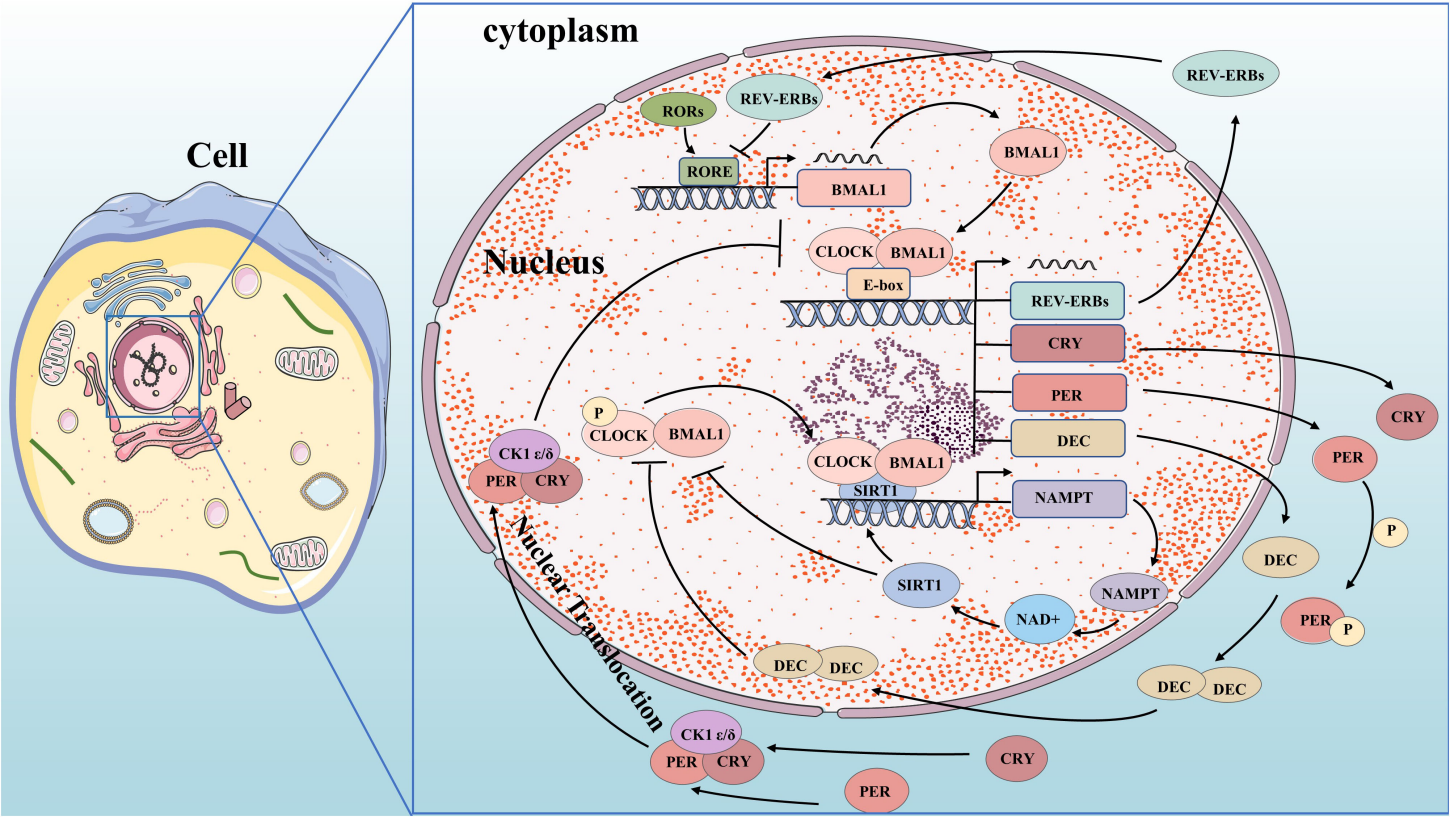
Molecular mechanisms of circadian clock. BMAL1 and CLOCK complex bind to the E-box and control gene promoters, including the genes for REV-ERBs, CRY, PER, and DEC. PER and CRY bind to CK1δ or CK1ϵ and translocate to the nucleus, where they inhibit their own transcription. DEC inhibits E-box promoters by directly binding to BMAL1 protein or CLOCK-BMAL1 complex and competing to suppress their expression. REV-ERBs compete for RORE in the promoter region of BMAL1 to inhibit its transcription. CLOCK-BMAL1 isoform can directly promote the circadian expression of NAMPT through the E-box of its promoter, thereby improving the circadian level of NAD+ and increasing the activity of SIRT1. Increased SIRT1 activity inhibits CLOCK-BMAL1 complex expression, forming a feedback loop that controls the circadian expression of core clock genes.

The Circadian clock is a regulatory system consisting of clock genes and controlled genes, which regulate the rhythmic movement of physiological and metabolic activities in organisms and synchronizes it with the changes in the environment. This regulation is mainly controlled by core transcription genes and their downstream regulated genes, which control the metabolic rhythm by regulating protein synthesis and degradation. The central clock system is located in the suprachiasmatic nucleus (SCN) of the hypothalamus, while the peripheral clock system is found in organs such as liver, spleen, lung, kidney, colon, adipose tissue and muscle. Light signals are converted into electrical signals by retinal ganglion cells and transmitted to the SCN through optic nerve tract to form rhythmic periodic activities that maintain organism homeostasis. Additionally, light signals can also be transmitted to the peripheral clock via neural and humoral pathways to synchronize them with external environment forming circadian rhythms (4). However, factors like body temperature, diet, and hormone levels also influence circadian oscillations (5–8). Before exploring the relationship between the circadian clock and lipid metabolism disorders as well as impact on cancer development, it’s important to understand how molecular mechanisms produce circadian oscillations within the circadian clock system (**Figure 1**).

The circadian oscillation is a self-regulating transcription-translation feedback loop (TTFL) that occurs periodically through the expression of circadian clock genes. Core clock genes, including ARNTL/BMAL1 and CLOCK as activators, and PER1,2,3 and CRY1,2 as repressors (9), are involved in this process. Transcription and translation of clock genes require cis-regulatory elements such as E-box, D-box, and RORE (ROR-elements). BMAL1 forms a heterodimer with CLOCK or NPAS2 to bind to the E-box regulatory elements on PER and CRY genes for transcription initiation (10–12). PER and CRY proteins form heterodimers in the cytoplasm which interact with casein kinase 1δ (CK1δ) and CK1ϵ kinases to undergo phosphorylation before translocating into the nucleus (13). In the nucleus, they combine with BMAL1-CLOCK heterodimers to form a quaternary complex that inhibits CLOCK-BMAL1 activity. This leads to reduced transcriptional activation of PER and CRY genes forming a negative feedback loop (14, 15). As CLOCK-BMAL1 complex activity is inhibited by this interaction, levels of PER and CRY proteins decrease. Once bound to F-box regulatory elements, specific E3 ubiquitin ligase complexes ubiquitinate PER and CRY proteins, leading to their degradation by the proteasome. This relieves inhibition on CLOCK-BMAL1 complex allowing for initiation of new rounds of transcription (15–17). The activity of BMAL1-CLOCK heterodimer is influenced by both ROR and REV-ERB proteins. BMAL1 and CLOCK activate REV-ERB by binding to its promoter region. In response, REV-ERB accumulates and competes with ROR for ROREs in the promoter region of BMAL1, inhibiting its transcription (18). Meanwhile, RORs compete to activate BMAL1 transcription, forming a secondary feedback loop. Additionally, the clock-controlled gene DEC (Deleted in Esophageal Cancer) forms an autoregulatory feedback loop through encoding basic helix-loop-helix (bHLH) transcription factors. DEC directly binds to the BMAL1 protein or CLOCK-BMAL1 complex and competes to suppress their expression by inhibiting E-box promoters (19–24). Clock genes play a crucial role in tumorigenesis by regulating oncogenes and tumor suppressors. We have started exploring the key signaling pathways in carcinogenesis based on the molecular mechanism of circadian clock genes.

### 2.2 Molecular mechanisms of lipid metabolism

**Figure 2:**
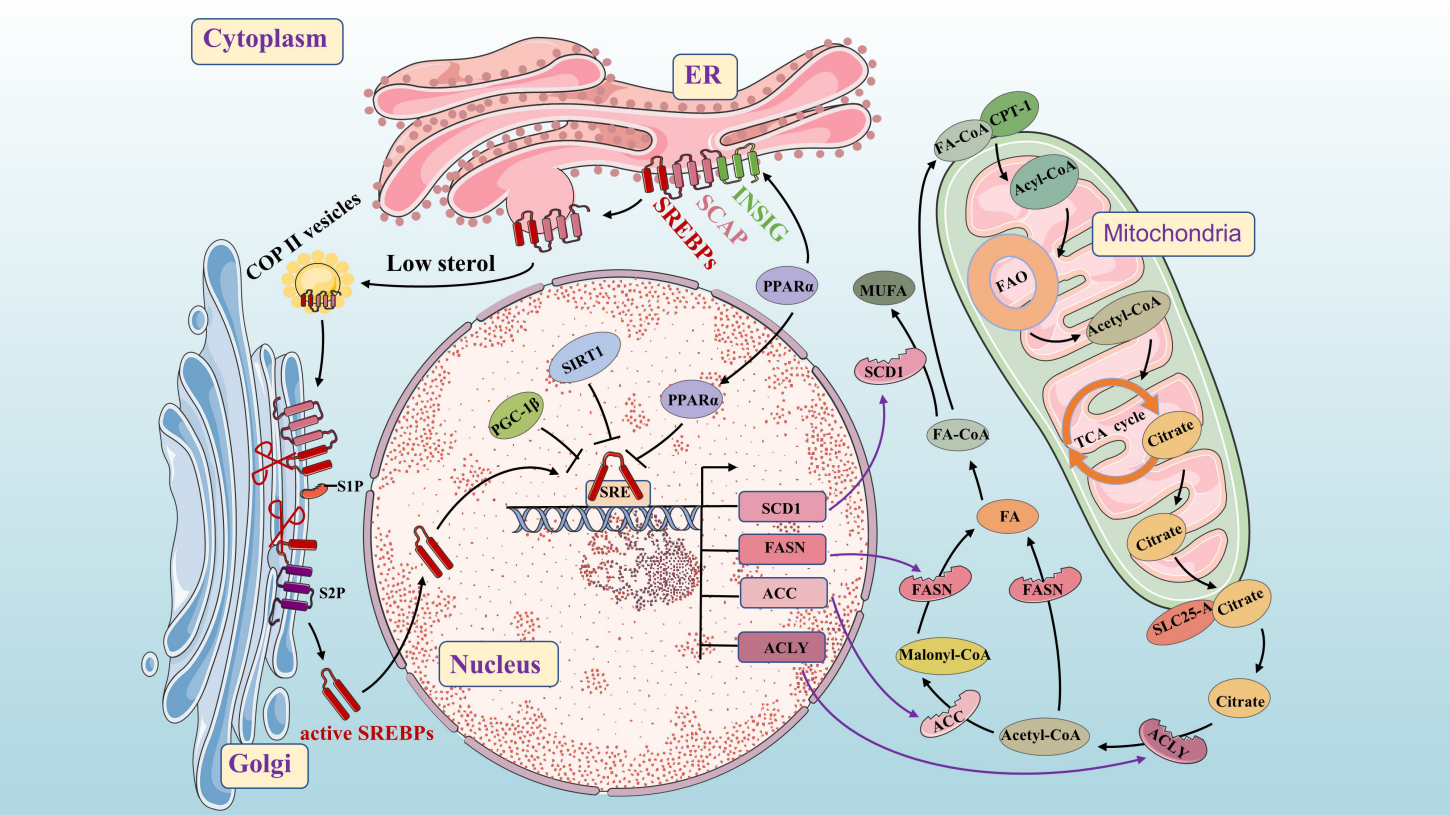
Molecular mechanisms of *de novo* fatty acid synthesis. As a key lipid source gene, SREBP is an important transcription factor throughout the process. The INSIG/SCAP/SREBPs complex is stable in the ER. When sterol levels decrease, SCAP dissociates from INSIG and facilitates the inclusion of SCAP/SREBP into COPII-coated vesicles for subsequent transport to the Golgi. PPAR α up-regulates the expression of the INSIG gene by binding to the PPRE in the promoter region and inhibits the nuclear translocation of the transcription factor SREBPs. SREBPs undergoes proteolysis under the action of S1P and S2P, and then translocate to the nucleus to activate SREBP target genes (such as FASN, SCD1, ACC, and ACLY). Citrate is acted upon by ACLY to form acetyl-CoA and oxaloacetate. Acetyl-CoA is converted into FA under the action of FASN. Acetyl-CoA is converted into Malonyl-CoA under the action of ACC, and then Malonyl-CoA is converted into FA under the action of FASN (Conversion of FA to MUFA by SCD). SIRT1 and PGC-1β can inhibit SREBP1 in the nucleus.

Lipids maintain cell structure, provide energy, and regulate cellular signaling. Lipid metabolism is regulated by various signaling pathways and produces different intermediates. Disturbances in lipid metabolism affect lipid levels, cell membrane composition, and permeability, thereby impacting the regulation of signaling pathways that contribute to cancer progression. Lipids consist of triacylglycerols (TAG), which are composed of fatty acids (FA) and glycerol, as well as adipoid. The majority of fatty acids in the body come from exogenous sources through food intake, while *de novo* FA synthesis contributes only a small proportion (25). Tumor cells enhance the *de novo* FA synthesis pathway to promote cancer cell biofilm formation and increase membrane lipid saturation. This alteration affects essential life processes such as cellular signaling and gene expression, supporting rapid cell proliferation and promoting cancer progression (26–28). Therefore, maintaining lipid homeostasis is crucial for human health, making research on lipid metabolism disorders an important focus in oncology. Understanding its molecular mechanism is necessary (**Figure 2**).

From previous studies, we know that Fatty acyl-CoA is transported across the mitochondrial membrane by carnitine palmitoyl transferase 1 (CPT1) and enter mitochondria for fatty acid β-oxidation (FAO), producing acetyl-CoA. Acetyl-CoA then enters the tricarboxylic acid (TCA) cycle to generate adenosine triphosphate (ATP) for energy production and combines with oxaloacetate to form citrate. Citrate is transported into the cytoplasm by a solute carrier family 25 member protein (SLC25-A), which is activated by SREBP1 (29). In the cytoplasm, citrate is converted into acetyl-CoA and oxaloacetate by ACLY. Under the action of ACLY, ACC and FASN, acetyl-CoA generates fatty acids in a closed circuit that regulate fatty acid metabolism in the body. The conversion of FA to monounsaturated fatty acids (MUFA) occurs through SCD (30). Cancer cells increase lipogenesis to promote their proliferation, and SREBP, a key transcription factor in FA synthesis, plays a central role in this process. It can affect the expression of ACLY, ACC, FASN and SCD1 at multiple stages of lipid biosynthesis (31, 32). SREBP consists of three subtypes: SREBP1a, SREBP1c and SREBP2 which regulate the expression of different lipid synthesis-related genes (33). SREBP1a and SREBP1c are encoded by the unigenes SREBF1 and mainly control the expression of lipogenic genes, while SREBP2, encoded by the unigenes SREBF2, primarily regulates cholesterol biosynthesis genes (34). Here, we focus on SREBP1. Previous studies have found that SREBP cleavage-activating protein (SCAP) binds to INSIGs protein in a sterol-dependent manner to form INSIGs/SCAP/SREBP1 complex stored in the endoplasmic reticulum (ER). At low sterol levels, SCAP dissociates from INSIG proteins and forms a complex with SCAP-SREBP1 that binds to coat protein complex–II (COP II) in the ER for translocation to the Golgi. In the Golgi, under the action of site 1 protease (S1P) and Site 2 protease (S2P), proteolysis occurs resulting in activation of nuclear translocation for increased FA biosynthesis through activation of target genes (35). When sterol content increases, changes occur in SCAP’s structure due to activation of its sterol sensing domain. This prevents entry into ER affecting vesicular transport from ER to Golgi and subsequently halts transcriptional regulation of FA synthesis (33, 36).

Peroxisome proliferator-activated receptor alpha (PPARα) is considered to be a crucial FA sensor (37). Its natural ligands include various FAs such as linoleic acid, oxidized fatty acids, and prostaglandin J2 (38, 39). PPARα mRNA is mainly expressed in tissues that oxidize fatty acids, such as the liver, heart, brown adipose tissue, kidney, and intestine (40). It regulates key transcriptional pathways involved in mitochondria, peroxisome and microsomal FAO as well as other lipolysis processes. Additionally, it plays a role in cellular functions like proliferation and metabolism. PPAR α mRNA is essential for maintaining nutrient homeostasis and lipid metabolism (38), while also regulating energy balance through activation of FA catabolism and stimulating of gluconeogenesis (41). Activation of PPARα increases the expression of acyl-CoA oxidase, CPT1, malonyl-CoA decarboxylase to enhance FA and triglycerides oxidation. Furthermore, PPARα activation down-regulates FASN and SREBP1 expression to impact *de novo* fatty acid synthesis (38, 42, 43). By binding to the PPAR response element (PPRE) in the promoter region, PPAR α up-regulates INSIG gene expression while inhibiting nuclear transfer of transcription factor SREBP1c to suppress FA production (44). Moreover, peroxisome proliferator-activated receptor γ coactivator β (PGC-1β), a transcription cofactor interacting with SREBP1c, can induce lipid synthesis-related gene transcription (45).

The activation of SREBP1c, a key transcription factor regulating lipid metabolism, in cancer cells and its impact on FA synthesis make it an important research focus for understanding tumorigenesis.

### 2.3 The relationship between circadian clock and lipid metabolism mechanism

**Figure 3:**
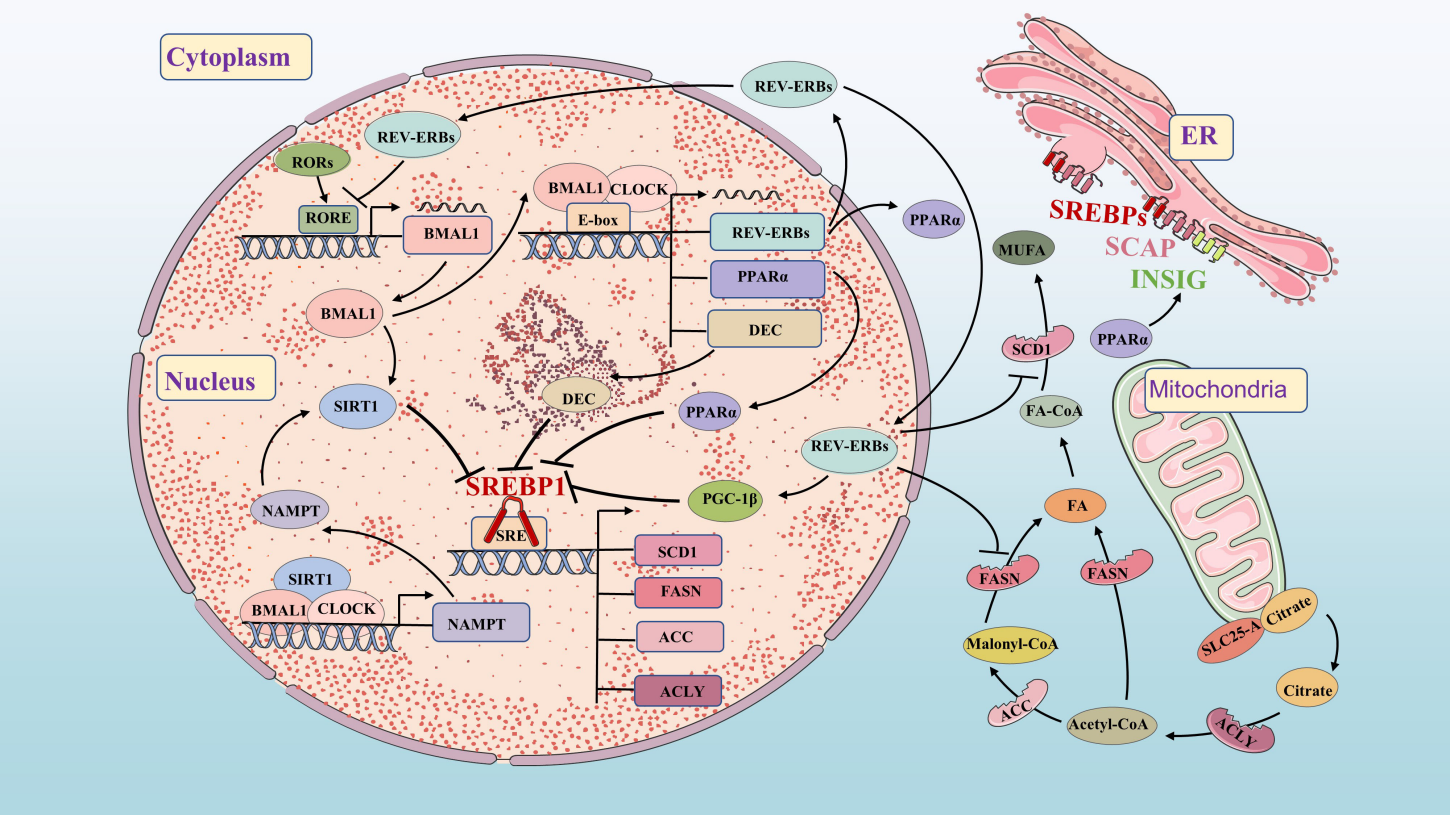
Molecular mechanisms of FA synthetic and circadian clock. FA synthetic and circadian clock networks have multiple interwoven negative feedback loops, such as BMAL1/DEC/SREBP1, PPARα/INSIG/SREBP1, BMAL1/SIRT1/SREBP1 and REV-ERBs/PGC-1β/SREBP1. The circadian network regulates the circadian expression of FA synthesis genes and metabolites through feedback loops.

The hypothalamus regulates the circadian rhythms of diet and energy metabolism, which in turn affect lipid metabolism through the active expression of metabolic enzymes and transport systems (46, 47). Lipids are primarily stored as TAG in the human body. Core clock genes control adipose lipolysis by regulating TAG levels (48). Deletion of both REV-ERBα and REV-ERBβ has been found to cause severe defects in lipid metabolism, including significant increased TAG levels in the liver and hepatic steatosis (49, 50). Numerous studies have shown that disruption in circadian clock function can lead to disorders in lipid metabolism (51). The circadian clock plays a crucial role in maintaining lipid homeostasis by rhythmically activating and regulating proteins involved in lipid transport, synthesis, and degradation (**Figure 3**).

Circadian variation in FA synthesis is partly mediated by the circadian clock’s effects on SREBP1c and its downstream targets (52, 53). Sirtuin 1 (SIRT1) deacetylates SREBP1c, inhibiting its activity and reducing occupancy on the promoter of lipogenesis genes. Increasing SIRT1 expression can enhance deacetylation of SREBP1c and suppress its expression, thereby inhibiting FA synthesis (54). SIRT1 is a NAD+-dependent histone deacetylase (HDAC) and a member of the HDAC family. Nicotinamide phosphoribosyltransferase (NAMPT) is the key enzyme of in NAD+ biosynthesis. The CLOCK-BMAL1 isoform directly promotes circadian expression of NAMPT, increasing the level of NAD+ and activating SIRT1. Increased SIRT1 activity inhibits CLOCK-BMAL1 complex expression, forming a feedback loop that controls circadian expression of core clock genes (55–57). DEC1 and DEC2 clock-controlled gene, regulate FA synthesis by inhibiting lipogenesis in the liver through binding to the promoter region of SREBP1c (58, 59). FASN and ACC also exhibit circadian fluctuations in adipose tissue and liver regulated by clock genes (60, 61). REV-ERBs effectively inhibit the expression of FASN, SCD1 and PGC-1β to impact FA synthesis (32, 62, 63).

PPARα directly binds to the circadian clock promoter, maintaining circadian oscillations of the BMAL1 gene in brain and muscle (64). BMAL1 regulates PPARα expression (65), while REV-ERBs is also a target gene of PPARα (66). The BMAL1-CLOCK complex activates PPARα by binding to the E-box (67). In turn, the response element of PPARα binds to the promoters of BMAL1 and REV-ERBs genes, activating their expression (65, 66). This highlights the close relationship between the circadian clock and FA metabolism. Previous studies have shown that both circadian clock and lipid metabolism disorders significantly impact carcinogenesis. Further research aims to understand key signaling pathways connecting circadian clock and lipid metabolism disorders in tumorigenesis, providing new directions for future investigations.

## 3 Effects of lipid metabolism transcription factors on cancer development

Although cancer types and genetic alterations vary, cancer is characterized by abnormal cell growth and proliferation, resulting in increased energy and macromolecules demands. These demands necessitate the synthesis of cellular building blocks such as nucleic acids, proteins, and lipids (68). Lipid synthesis is particularly enhanced in malignant tumors (68, 69), contributing to their aggressiveness. In fact, lipid synthesis is generally upregulated in human cancers (70), with 95% of tumor cell fat derived from endogenous *de novo* lipogenesis compared to normal cells (71). Notably, the key transcription factor SREBP1 and its downstream lipid-derived genes ALCY, ACC, FASN, and SCD1 play crucial roles across various tumors (**Table 1**).

**Table 1:** Association of *de novo* fat synthesis genes with tumor development.

Factor	Notes	Disease	Proposed function	References
**SREBP1**	Downregulation	Ovarian cancer (OV), Clear cell renal cell carcinoma (ccRCC), Pancreatic cancer (PAAD)	Anti-proliferative, anti-migration, anti-invasion and pro-apoptotic	(72–75)
		Non–Small-Cell Lung Cancer (NSCLC)	Reduce chemotherapy resistance and acquired resistance	(76–79)
	Upregulation	Breast cancer (BRCA)	Enhanced proliferative, migration and invasion	(80)
**SCD1**	Downregulation	Prostate cancer (PRAD), BRCA, Hepatocellular carcinoma (HCC), Endometrial cancers (EC)	Anti-proliferative and anti-invasion.	(30, 81–85)
	Upregulation	Nasopharyngeal carcinoma (NPC), Colorectal Cancer (CRC), (Gastric cancer) GC	Pro-proliferative and pro-migration, poor prognosis.	(86–88)
		HCC	Enhance drug sensitivity	(89, 90)
**FASN**	Downregulation	NSCLC, EC, Esophageal squamous cell carcinoma (ESCC), CRC, PRAD	Anti-proliferative, anti-migration, anti-invasion and pro-apoptotic.	(91–98)
		NPC	Enhanced radiotherapy sensitivity	(99)
	Upregulation	NPC	Pro-proliferative, pro-invasion and pro-migration,	(100)
**ACLY**	Downregulation	PRAD, NPC, HCC	Anti-proliferative, anti-migration and pro-apoptotic.	(101–104)
		BRCA	Improve curative effect	(104)
		OV	Reduce chemotherapy resistance.	(105)
		HCC	Enhance cancer stem cell.	(106, 107)
	Upregulation	HCC, PRAD	Pro-proliferative, pro-invasion and pro-migration	(108, 109)
**ACC**	Downregulation	NSCLC, HCC	Anti-proliferative, anti-migration and pro-apoptotic.	(110–112)
	Upregulation	PRAD	Enhanced hormone resistance	(113)
**PPAR**	Downregulation	BRCA, NSCLC,	Shortened survival and pro-migration	(114, 115)
		RCC, BRCA, lung cancer	Anti-proliferative, anti-migration and pro-apoptotic	(116–119)
	Upregulation	HCC	Pro-apoptotic	(120)

### 3.1 SREBP1

SREBP1 primarily controls the expression of lipogenic genes (121). It acts as a key transcription factor regulating *de novo* lipogenesis and lipid homeostasis. In tumor cells, SREBP1 plays a crucial role in promoting growth, survival, proliferation, apoptosis, invasion and metastasis (72, 73, 122, 123). Overexpression of SREBP1 is closely associated with cancer progression and metastasis. Conversely, knockdown of SREBP1 reduces the expression of FA synthesis genes, inhibits cancer cell proliferation, and suppresses tumor growth (124). SREBP1 plays a significant role in promoting lipogenesis and tumor growth in breast cancer (BRCA), hepatocellular carcinoma (HCC), Esophageal squamous cell carcinoma (ESCC), pancreatic cancer (PAAD), and gastric cancer (GC) (74, 80, 125). Inhibition of SREBP1 can hinder tumor progression and metastasis. SREBP1 also plays a role in chemotherapy resistance among cancer cells which has been associated with reduced patient survival rates. Targeting SREBP1 can inhibit lipid synthesis, gemcitabine-induced CSCs, increases sensitivity to gemcitabine, reducing chemical resistance, and ultimately improving patient survival rates (75, 126, 127). In addition to chemical resistance, Osimertinib facilitated degradation of mature form of SREBP1 and reduced protein levels regulated by genes in EGFR-mutant NSCLC. This inhibition leads to decreased lipogenesis, cancer cells apoptosis, and reduced acquired resistance to Osimertinib (76). Moreover, inhibition of SREBP1 results in lipid peroxidation. Patients with EGFR-mutated NSCLC developed resistance to gefitinib following targeted therapy. However, SREBP1 restoration restores their sensitivity to gefitinib and exhibits synergistic effects on anti-proliferation and pro-apoptosis (77–79). In conclusion, SREBP1 is generally up-regulated in CRC, HCC, PRAD, BRCA, THCA, NSCLC and PAAD, which aligns with its role in regulating lipid homeostasis in human cells.

### 3.2 SCD1

SCD1, a rate-limiting enzyme in MUFA biosynthesis, is overexpressed in lung cancer, BRCA and OV (128–132). Inhibiting SCD-induced accumulation of MUFA leads to cancer cell death (133), and SCD1 has also been identified as a marker of CSCs in CRC (134). Increased expression of SCD1 is associated with tumor progression and poor prognosis (30, 135). Its expression is regulated by factors such as the millet bran (a total polyphenol consisting of 12 compounds extracted from foxtail millet bran, BPIS), Hypoxia-inducible factor-1α (HIF-1α), and miR-433-3p. Targeting SCD1 may be a potential therapeutic strategy for different cancers (81, 82, 86). Cancer cells rely on regulating SREBP1 expression as well as activating both SCD1 and FASN to promote lipogenesis and proliferation (87, 88). In genetic and pharmacological studies, inhibiting SCD1 alters cellular lipid composition which disrupts plasma membrane fluidity leading to inhibiting of HCC cell invasion *in vitro*, which could serve as a biomarker for HCC aggressiveness (30, 83). Studies on FA metabolism in EC have shown that reducing SCD1 expression can inhibit EC growth (84). SCD1 has been associated with drug resistance acquisition. The multikinase inhibitor sorafenib targets SCD1 through the ATP-AMPK-mTOR-SREBP1 pathway to suppress MUFA synthesis, disrupt lipogenesis, induce liver cancer cell death, and enhance sensitivity to sorafenib (89, 90) Docetaxel effectively impedes the tumor progress in PRAD by down-regulating mRNA and protein levels of SREBP1 and SCD1. This enhances docetaxel’s anti-proliferation, anti-migration and anti-invasion capabilities (85).

### 3.3 FASN

FASN up-regulation promotes cancer progression by enhancing lipid synthesis and signaling pathways, while its inhibition can impede tumor development and indicate poor prognosis. Compared to normal human tissues, increased FASN expression promotes endogenous FA synthesis in various cancer tissues (91–94, 136–138). FASN is associated with tumor invasiveness, and increased expression of FASN is positively correlated with lipid droplet formation and enhanced cancer cell activity. FASN regulates metabolic disorders and can indicate poor prognosis (139–142). Various compounds have been found to reduce FASN expression and inhibit lipogenesis, demonstrating potential anticancer activity such as extract of eriobotrya japonica and Davallia formosana, Oridonin (95–97). Additionally, Long intergene non-coding RNA2570(LINC02570) promotes NPC progression by up-regulation SREBP1 and FASN through miR-4649-3p (100). In the reprogramming of lipid metabolism that occurs in cancer-associated fibroblasts (CAF), FASN is significantly increased in CAF, enhancing colorectal cancer cell migration (98, 143). Moreover, FASN inhibitors can enhance the effects of chemotherapy drugs, restore tumor sensitivity to treatment, and inhibit tumor growth in resistant cancer cells (144, 145). Combination therapy with FASN inhibitors and other targeted therapies or radiotherapy shows promising therapeutic effects (146). Additionally, some studies have demonstrated that inhibiting the FASN gene significantly increases nasopharyngeal carcinoma cell sensitivity to radiotherapy (99).

### 3.4 ACLY

ACLY is up-regulated in various tumors and plays a crucial role in cancer cell proliferation, growth, migration and apoptosis (147–149). Its overexpression provides energy for malignant proliferation of tumor cells and promotes their progression (101). Increased expression of ACLY and SCD1 mRNA in HCC leads to enhanced FA synthesis, resulting in cell proliferation and deterioration of HCC (108). Modulating the expression of ACLY and FASN through SREBP1 affects *de novo* lipogenesis production in PRAD cells, promoting cell proliferation (109), which is consistent with the inhibitory effects observed upon ACLY knockdown in NPC and PRAD that inhibit tumor cell migration and growth (102, 103). Targeting ACLY can synergistically enhance the efficacy of targeted therapy and chemotherapy while reducing drug resistance. The ACLY inhibitor BMS-303141 induces apoptosis in HCC cells when combined with sorafenib to improve therapeutic efficacy (101). Competitive inhibition of ACLY by Hydroxycitric acid enhances tamoxifen’s toxic effect on breast cancer cells, improving its efficacy (104). Down-regulation of ACLY promotes apoptosis in ovarian cancer cells while attenuating cisplatin resistance (105). Increased levels of FASN and ACLY contribute to cancer stem cell-like properties, self-renewal induction, cellular steatosis, affecting HCC progression (106, 107). Additionally, ACLY has emerged as a potential biomarker for predicting breast cancer recurrence (150).

### 3.5 ACC

ACC plays a crucial role in *de novo* lipogenesis and its inhibition can hinder tumor nutrient supply and development, making it a potential target for cancer treatment. It has been reported that FASN utilizes ACC for *de novo* lipogenesis (151). The regulation of *de novo* lipogenesis affects the tumor’s energy supply and is divided into two phenotypes: ACC1 (ACCα or ACACA) and ACC2 (ACCβ or ACACB) (152). ACC1 is highly enriched in lipid tissues, while ACC2 occurs in oxidized tissues and has distinct metabolic effects due to their different locations (153). Malonyl-CoA produced by ACC1 serves as a substrate for lipogenesis, whereas malonyl-CoA produced by ACC2 inhibits CPT1, thereby preventing FA degradation. Upregulation of ACC1 has been observed in various tumors, likely promoting lipogenesis to meet the demands of rapid growth and proliferation (70, 154, 155). Therefore, it can be speculated that inhibiting ACC expression can hinder tumor nutrient supply and tumor development. Knockdown of SREBP1-associated genes such as ACLY and both isoforms of ACC in NSCLC cell lines promote cell apoptosis and differentiation (110, 111). Moreover, reduced phosphorylation of ACC was found to increase HCC genesis in mice and the proliferation of liver cancer cells. Inhibitors targeting allosteric sites on ACC can alleviate HCC deterioration and improve survival rate (112). Other studies have highlighted the significance of ACC and FASN expression in hormone resistance and cancer prevention (113).

### 3.6 PPAR

PPARα plays a crucial role in lipid metabolism and is highly expressed in organs with significant FA catabolism, such as the liver (156). It acts as key regulator of lipid and glucose metabolism, controlling FA catabolism and lipoprotein metabolism (157). As a ligand-activated transcription factor, PPAR is involved in cellular processes like cell differentiation, proliferation, and apoptosis (158–160). When activated, PPARα exhibits antiangiogenic and anti-inflammatory effects, thereby inhibiting tumor development (161). PPARα agonists like fenofibrate and WY-14,643 have been found to inhibit tumor growth in multiple cancer studies and are potential targets for treating various malignancies (38). Knockdown of PPARα has been associated with reduced breast cancer-specific survival (114). The chemical sensitivity of breast cancer cells to the PPARα agonist clofibrate was high. Clofibrate significantly affected the PPARα DNA-binding activity and free FA production while effectively reducing levels of SREBP-1c and FASN. It targeted lipogenic and inflammatory pathways in invasive breast cancer (162). The PPARα agonist clofibrate induced apoptosis in HepG2 cells in both time-dependent and concentration-dependent manners (120).

Interestingly, PPARα regulates FAO activity to meet the higher energy requirements of high-grade renal cell carcinoma (RCC) compared with low-grade RCC (163). Additionally, in PAAD and CRC, the PPARα signaling pathway ensures high lipid turnover in cancer stem cells, maintaining their high energy requirements and self-renewal (164). The levels of PPARα protein were associated with RCC invasiveness in two renal cell carcinoma cell lines (AKI-1 and 786-O) (116). Furthermore, loss of PPARα expression in host animals inhibited tumor growth in lung cancer cells according to a study using a mouse xenograft model (117). Studies have demonstrated that regulating the PPAR/NF-κB signaling pathway can promote multi-organ distant metastasis of NSCLC (115). Mice lacking PPARα showed resistance to increased DNA synthesis and liver tumorigenesis induced by the agonist WY-14, 643, further supporting the involvement of PPARα in HCC (165). In breast cancer stem cells, the PPARα antagonist GW6471 has anti-proliferation and pro-apoptotic effects while the PPARα agonist Wy14643 promotes clonal expansion through NF-κB/IL-6 axis signaling activity promotion (118, 119). Clofibrate was also found to promote OV and PRAD progression without correlation with PPARα (166). Therefore, it is evident that the influence of PPARα on tumor progression varies depending on tissue type and difference in its ligand. This highlights the potential for developing specific synthetic ligand targeting PPARα as a novel approach for cancer treatment.

## 4 Influence of circadian clock genes on cancer development

Circadian rhythm disorders are independent risk factors for cancer, as disruption of the circadian clock may be associated with cancer cell proliferation, senescence, metabolism and DNA damage (167–169). However, the exact molecular mechanisms underlying this effect have yet to be elucidated. Studies have shown that disturbance or dysregulation of circadian rhythm is associated with poor prognosis of various tumors. CLOCK genes such as BMAL1 and REV-ERB mainly play an anticancer role while DEC has both anticancer and cancer-promoting effects in relevant literature (**Table 2**).

**Table 2:** Association of circadian rhythm genes with cancer development.

Factor	Notes	Disease	Proposed function	References
**BAML1**	Downregulation	NSCLC、BRCA	Enhanced proliferative, migration and invasion	(170–172)
		PAAD	Poor prognosis.	(173)
	Upregulation	CRC	Enhanced drug sensitivity.	(174)
		PAAD	Anti-proliferative and anti-invasion	(175)
		BRCA	Pro-migration and pro-invasion	(176)
**REV-ERB**	Upregulation	Cervical Cancer, Esophageal cancer (ESCA), BRCA, Small-Cell Lung Cancer (SCLC)	Anti-proliferative and pro-apoptotic	(177–179)
**DEC**	Downregulation	OV	Anti-proliferative, anti-migration, anti-invasion and pro-apoptotic	(180)
		NSCLC	Promoter tumor progress	(181)
	Upregulation	NSCLC, CRC	Pro-proliferative and anti-apoptotic	(172, 182)
		BRCA, ESCA	Anti-proliferative and pro-apoptotic	(183, 184)
		Cervical Cancer, GC, CRC	Anti-apoptotic	(185–187)

### 4.1 BAML1

The circadian clock is composed of rhythmic genes and gene products that regulate the expression of clock control genes, generating a distinct circadian rhythm. BMAL1 serves as a crucial transcription factor within the transcription-translation feedback loop of the circadian clock. Its functions are associated with cellular processes such as metabolism, proliferation, metastasis, and cell cycle regulation. Additionally, it plays a significant role in modulating oncogenes and tumor suppressor genes (175, 188). Analysis conducted on GSE39582, GSE21510 and Cancer Genome Atlas (TCGA) pan-cancer datasets revealed notable down-regulation of BMAL1 expression in BRCA and CRC tumor samples when compared to normal tissues. However, in tumors exhibiting TGFβ activation or BRAF mutations, BMAL1 showed slight up-regulation (189, 190). Knockdown of BMAL1 disrupts circadian rhythm and enhances migration or invasion in lung cancer, breast cancer, and glioma cells (170, 171). Loss of BMAL1 may contribute to lung cancer development by activating KRAS and cancer-regulating genes such as P53 and c-Myc (172). Overexpression of BMAL1 inhibits proliferation and increases sensitivity to oxaliplatin in CRC cell lines and HCT116 cell models *in vivo* (174). Immunohistochemical analysis reveals that low Bmal1 expression in tumor tissues significantly impact tumor progression and prognosis compared to adjacent non-tumor tissues (173). *In vitro* experiments demonstrated that BMAL1 overexpression suppresses proliferation and invasion of pancreatic cancer cells through the activation of P53 pathway (175). Interestingly, on the contrary, BMAL1 overexpression promotes invasion and metastasis of breast cancer cells by upregulation matrix metalloproteinase 9 (MMP9), a mediator of local tumor invasion and distant metastasis (176). Additionally, CLOCK and BMAL1 overexpression can promote cancer cells growth by affecting F-actin formation (191), indicating that regulation of BMAL1 has diverse effects on tumor proliferation, invasion and metastasis across different oncogenic pathways.

### 4.2 REV-ERB

The nuclear hormone receptors REV-ERBα and REV-ERBβ (REV-ERBs) are important components of the circadian clock (49, 50). Abnormal expression of REV-ERBs has been observed in various cancer types and is involved in tumor metabolism, proliferation, as well as regulation of plasma glucose levels, lipid and energy metabolism (192–195). Treatment with agonists SR9011 and SR1078 specifically enhances the function of REV-ERBα and RORα receptors in cells. After 72 hours of agonist treatment, cervical cancer and esophageal cancer cells exhibit dose-dependent decreases in proliferation accompanied by induced apoptosis. Moreover, these agonists have minimal impact on the viability of normal cells or tissues (177). In breast cancer cells, SR9011 remains unaffected by ER and HER2 expression while inhibiting the proliferation of breast cancer cell lines (178). The REV-ERB agonist SR9009 selectively induces cell death in both chemosensitive and chemoresistant SCLC cells. REV-ERBα demonstrates antitumor effects in SCLC cells (179). Activation of REV-ERBα eliminates oncogene-induced senescent cells, mediates chemotherapy resistance and relapse, thus making REV-ERBα agonists potential therapeutic options for different types of cancer (62).

### 4.3 DEC

The DEC family genes, including DEC1 and DEC2, are expressed by differentiated embryonic chondrocytes. Physiologically, DEC1 and DEC2 are both expressed in a circadian manner and are regulators of the mammalian circadian clock, forming the fifth clock gene family that regulates circadian and metabolic functions (19, 196, 197). Abnormal expression of DEC1 is associated with tumor development and invasiveness, making it a potential predictor for cancer prognosis after treatment (183, 198, 199). Dysregulated expression of DEC may alter normal circadian rhythms and significantly contribute to the development of various diseases, including cancer. It has been observed that DEC1 expression is increased in various cancers and promotes cell proliferation and survival, while DEC2 expression is low in lung cancer. The impact of DEC on tumor development varies depending on the specific cancer type (23, 172, 180–182, 185, 200). Furthermore, it has been found that the role of DEC1 in apoptosis may differ among different type of cancer tissues. DEC1 exhibits a pro-apoptotic effect in BRCA and ESCA (183, 184). But it has an anti-apoptotic effect in cervical cancer, GC, and colon cancer (185–187). Currently, the effect of DEC on tumor remains controversial; however, there is no doubt that alterations in the level of the DEC gene significantly influence tumor occurrence and progression.

Circadian clock genes have a dual role in cancer, acting as tumor suppressors in most cases but possibly serving as catalysts in specific cancer cells. The precise mechanisms and factors governing their roles as oncogenes or tumor suppressors are still unknown, posing both challenges and opportunities for future research.

## 5 The influence of interlocking of circadian clock and lipid metabolism on cancer

Circadian clock disorders strongly affect tumor transformation and tumor growth by altering a variety of cancer regulatory pathways, such as lipid metabolism. Multiple organs of the human body are involved in lipid metabolism, and these organs are controlled by the circadian clock to regulate the body’s metabolic functions. In metabolic organs such as white adipose tissue, liver, gut, pancreas and muscle, the circadian clock drives the rhythmic expression of export genes involved in metabolism, biosynthesis, signal transduction, and cell-cycle pathways. It also coordinates glucose, lipid, and protein metabolism (201). Researchers have found a link between the circadian clock regulated by chromatin remodeling and cellular metabolism, suggesting that metabolic disorders in cancer may be the result of circadian clock disturbances (169, 202). Cancer cells use metabolic reprogramming to meet the energy requirements for rapid cell proliferation and survival, indicating metabolic plasticity. Mitochondria contain metabolic centers that catabolize fatty acids, amino acids, and glucose to provide energy for cell growth. Related studies have found that circadian gene expression and mitochondrial activity seem to interact and balance each other, but the detailed underlying mechanisms are still unclear. Previous studies have found a link between cancer cells’ metabolic disorders and disruptions to the circadian clock, which may play an important role in cancer progression (203).

50% of liver metabolites in mice are regulated by circadian rhythm (204). The circadian rhythms of 50% of metabolites in liver, muscle, adipose tissue, and serum were phase shifted in mice fed a high-fat diet (61, 205–207). The disruption of the circadian system leads to the dysregulation of chronic jet lag driver genes, resulting in hepatic metabolic dysfunction and changes in circulating energy consumption, lipid metabolism, insulin, and glucose signaling to promote the occurrence of liver cancer (204, 208). Systemic and liver-specific knockdown of BMAL1 can cause metabolic disorders, leading to hyperlipidemia and increased lipoprotein production (209). In untreated MDA-MB-231 cells and chronically insulin-treated MDA-MB-231 cells, BMAL1 knockdown inhibited the utilization of glutamine and FA by increasing their oxidation (189). Disruption of circadian clock genes can cause disorders of lipid metabolism. BMAL1 can play a role as a tumor suppressor in obese mice, inhibiting the growth of BRCA and lung metastases, and the down-regulation of BMAL1 was associated with an increased risk of breast cancer metastasis (189). It has been reported that BMAL1 can regulate metabolic reprogramming and affect the expression of PD-L1 in macrophages (210), suggesting that the circadian clock influences tumor development by regulating metabolic pathways.

REV-ERBα is a transcription factor that plays an important role in a series of physiological processes, including the regulation of glucose, lipid metabolism, and circadian rhythm, as a core inhibitory component of the cell autonomous clock and a regulator of metabolic genes (194, 211–213). REV-ERBα and β are present on several key lipid and bile acid regulatory genes, including Insig2 and SREBP, providing mechanisms for rhythmic lipid and bile acid metabolism molecules (50). Coordination of REV-ERBα and REV-ERBβ activities is required for normal hepatic clock gene expression and lipid metabolism (49). The hepatic circadian clock regulates the transcription function of the circadian transcription suppressor REV-ERBα, thereby controlling the production of SREBP-dependent fatty acids, cholesterol, and bile acids (53). REV-ERBs can also inhibit lipid producing enzymes including FASN and SCD1, and strictly control lipid metabolism (63). When comparing metabolic parameters between tamoxifen-treated controls and REV-ERBα and β double knockout animals, it was found that the double knockout mice showed increased circulating glucose and triglyceride levels and decreased free fatty acid levels (50). Cancer cells are highly dependent on *de novo* lipogenesis, which plays a central role in meeting the metabolic needs of cancer cells and is one of the important cancer markers involved in the anticancer activity of REV-ERB agonists (1). REV-ERB agonists SR9009 and SR9011 play a key role in regulating autophagy and *de novo* lipogenesis to induce an apoptotic response in malignant cells. REV-ERB agonists can act as novel inhibitors of *de novo* lipogenesis and have selective activity against malignant and benign tumors (62).

SIRT1 affects the circadian expression of core clock genes BMAL1 and ROR γ, which in turn is controlled by NAD-dependent mammalian sirtuins (214–217). The levels of NAD, NADP, NADH, and NADPH affect the ability of CLOCK-BMAL1 heterodimer to bind to E-box elements (218). Meanwhile, the circadian clock can regulate the rhythmic activity of niacinamide phosphoribosyl transferase (NAMPT), thus regulating the cyclic availability of NAD and forming a closed pathway (56, 57). SIRT1 can not only modify the expression rhythm of circadian clock genes (CLOCK and BMAL1) but also affect the expression rhythm of clock-controlled genes related to lipid metabolism (PPARα, SREBP-1c, ACC1, and FASN) in high-fat diet mice (219). SRT1720 (a chemical activator of SIRT1) inhibits the expression of SREBP target genes, and orthologs of SIRT1 inhibit lipid synthesis and fat storage by downregulating SREBP orthologs during fasting (220). SIRT1 has also been shown to alter cellular metabolism and responses to stress, thereby affecting the progression of direct transcription, apoptosis, autophagy, DNA damage repair, and senescence (221–223). SIRT1 overexpression has been found in BRCA, PRAD, GC, CRC, and liver cancer (224–228). Significant upregulation of SIRT1 promotes tumor proliferation, migration, and invasion by targeting SREBP1 and lipogenesis in EC (229). SIRT1 expression has been found to be significantly associated with distant metastatic recurrence and reduced survival in BRCA (224). Down-regulation of SIRT1 can continuously inhibit the proliferation of HCC and prostate cancer cells by inducing senescence or apoptosis (226). SIRT1 can inhibit PRCA invasion and enhance chemical sensitivity (230).

Through the above studies, it has been found that SIRT1 functions as an oncogene, and the inhibition of SIRT1 expression can inhibit tumor development. However, some studies have found that SIRT1 acts as a tumor suppressor gene in cancer tissues. After the downregulation of NAMPT expression, the activity of SIRT1 deacetylase significantly decreased, and the gene expression of two key lipid factors, FASN and SREBP1c, significantly increased, promoting the accumulation of triglycerides in HepG2 cells (231). SIRT1 mRNA was found to be down-regulated in GC (232), which is significantly related to shortened overall survival and relapse-free survival in gastric cancer (233). SIRT1 depletion enhanced proliferation and metastasis, promoting the growth of GC. SIRT1 may play a role as a tumor suppressor (228). However, SIRT1 overexpression inhibits lipid metabolism in prostate cancer cells by activating AMPK phosphorylation and inhibiting SREBP1 expression and nuclear translocation. Additionally, astragalus polysaccharide has been found to inhibit tumor progression and lipid metabolism by regulating the miR-138-5p/SIRT1/SREBP1 pathway (234). The present results suggest that SIRT1 has dual roles as a tumor promoter and tumor suppressor (235).

PPAR is involved in circadian clock control independently of the suprachiasmatic nucleus (236). CLOCK and BMAL1 play important roles in lipid homeostasis by regulating the circadian activation of controlled target genes of potential PPAR response elements (237). PPARα deficiency disrupts the normal circadian regulation of certain SREBP-sensitive genes in the liver (238). Furthermore, studies have shown that the PPARα-SCD1 axis promotes CSC properties in HCC cells. Inhibition of the PPARα pathway or SCD1 decreases the expression of CSC-related markers, leading to the loss of CSC properties (239).

From the above studies, it is reasonable to speculate that the circadian clock may be related to increased *de novo* fatty acid synthesis in tumors, and tumor-dependent metabolites may be secreted in a temporal manner, which indicates that targeted pharmacological studies can be conducted on the daily peak of fatty acid metabolism pathway. Although the specific mechanism of the occurrence and development of cancer is still unknown, a large number of studies have been conducted on the related role of circadian clock and lipid metabolism in the process of tumor development. How circadian clock affects tumors by regulating lipid metabolism has been explored, but still needs to be further studied. Collectively, there is a key signaling axis involved that coordinates the central pacemaker and peripheral circadian transcription with lipid metabolism, although the implications of these findings for humans remain unclarified.

## 6 Cancer therapeutic strategies based on circadian clock and lipid metabolism

### 6.1 Targeting lipid metabolism disorders

In the reprogramming of lipid metabolism in cancer cells, endogenous FA are usually up-regulated and are essential for maintaining cell proliferation, division, and ATP energy (240). FA are substrates for producing lipid signaling molecules, and the mutual adjustment between lipid metabolic factors and oncogenic signals affects tumor proliferation, migration, and apoptosis. There is a close relation between the abnormal increase of *de novo* fatty acid synthesis and the growth and differentiation of cancer cells. SREBP1, ACLY, ACC, FASN, SCD and PPARα have been widely studied as key lipid metabolic factors. SREBP regulates lipid homeostasis by controlling the expression of a series of enzymes required for the synthesis of endogenous FA, cholesterol, triacylglycerol, and phospholipids. SREBP inhibitors reduce cell membrane fluidity, which leads to decreased tyrosine phosphorylation of EGFR and enhances the sensitivity of gefitinib in lung cancer cells (79). ACLY provides energy for cancer cells to function properly during catabolism and biosynthesis. ACLY overexpression in a variety of cancers indirectly destroys citrate binding by altering the citrate binding site of the enzyme, which is one of the options for cancer treatment. The ACLY inhibitor SB-204990 was found to inhibit the growth of tumors, such as lung cancer and PRAD (241). In addition, the functional polymorphism of ACLY can also be used as a prognostic marker to predict the recurrence of CRC (242). ACC is a key enzyme in the process of tumor lipid metabolism. It was found that ACC inhibitor ND-654 inhibited the proliferation and differentiation of liver cancer cells by inhibiting the production of nascent FA (112). Preclinical animal studies have shown that ACC inhibitors ND-646 and ND-654 significantly inhibit the growth of mouse lung tumors and rat HCC, respectively (110, 112). Additionally, ND-646 also inhibited the growth of NSCLC (243). TVB2640, a FASN inhibitor, has been observed to have a significant inhibitory effect on tumor growth in cancer cell lines and xenograft models, but due to its pharmacological nature, its clinical transformation and application are limited (244). In ovarian cancer models, the SCD1 inhibitor BZ36 reduces SCD1 expression and increases cancer cell sensitivity to ferroptosis inducers, thereby inducing tumor cell apoptosis (245). One study found that activators of PPARα could be used to prevent or treat CRC (246). In a variety of cancer types, a considerable number of drugs that can inhibit the synthesis of SREBP, ACLY, ACC, FASN, SCD, and PPARα have been tested for anticancer effects in preclinical and clinical studies. Targeting lipid metabolism has been confirmed to have positive anti-tumor effects, which may become a new therapeutic strategy for cancer.

### 6.2 Chronotherapy as a new therapeutic strategy

The circadian clock regulates the absorption, distribution, metabolism, and elimination of drugs (247). Circadian timing of drugs may be an important parameter in the treatment of diseases, therefore, chronotherapy has received a lot of attention. Chronotherapy is a strategy that uses the natural rhythms and cycles of the physiological and biochemical processes of an organism to treat disease (248–250). Prior to the discovery of a more detailed mechanism of the core clock, therapeutic strategies to reduce toxic side effects and improve efficacy during cancer treatment by controlling the duration of treatment have been used in clinical practice (251–253). It has also been found through mechanistic studies that many anticancer drugs have been shown to increase cytotoxicity at specific stages of cell division (247, 254). Chronotherapy was also found to improve survival and quality of life in cancer patients by minimizing the cytotoxicity of anticancer drugs (255–257). This suggests that optimizing the timing of treatment administration by predicting the drug properties associated with circadian rhythms can translate into desired clinical outcomes. It has also been shown that for anticancer drugs that are limited by their ability to cause side effects on healthy host tissues, regulating the timing of administration in accordance with their circadian characteristics not only helps to produce beneficial effects, but also avoids adverse effects (247, 258, 259). Multiple phase I to III clinical trials have demonstrated the effectiveness of chronotherapy in various tumors (260, 261). More than 30 chemotherapeutic drugs were found to differ in efficacy by more than 50% due to the different time of administration (259). One study, which included 186 cases of patients with metastatic colorectal cancer in a randomized multicenter phase III trial, found that compared with the constant rate of infusion, adjusting the delivery time of oxaliplatin, 5-fluorouracil (5-FU), and folic acid reduced the incidence of severe mucosal toxicity by about 5 times. At the same time, the rate of peripheral nerve lesions caused by dysfunction decreased by about 50% (251). Additionally, it was found that the maximum plasma concentration and optimal tolerance time were at 4:00 a.m. in cancer patients treated with 5-FU for 5 days (262, 263). In addition, timed irinotecan therapy in 31 cancer patients showed that time-mediated irinotecan infusion caused less diarrhea and patient-to-patient variability than the traditional 30-minute morning infusion (264). In a study of 41 patients with NSCLC, lower gastrointestinal toxicity was observed in patients treated with cisplatin at 6 p.m. or 6 a.m. compared with conventional chemotherapy, and a higher clearance rate was observed in patients treated with cisplatin at 6 p.m (265).. The REV-ERBs agonists SR9009 and SR9011 have been reported to have anticancer activity in different tumor types, including leukemia, brain, colon, breast, and melanoma, without significant side effects on normal cells or tissues (62, 178, 179, 266). In addition, autophagy and *de novo* lipogenesis were identified as key events in initiating apoptotic responses in malignant cells treated with SR9009 and SR9011 (62). SR9011 and SR9009 also reduce the proliferation of glioblastoma stem cells by inhibiting the expression of TCA cycle enzymes and inhibiting autophagy, and are lethal to chemoresistant small cell lung cancer cells (179, 267). However, paradoxically, the study found that SR9009 can exert an effect on cell proliferation independently of REV-ERB proteins, questioning the role of REV-ERB in the action of the drug specifically in cancer treatment (268).

A comparison of the morning and evening doses of cisplatin in patients with prostate, breast, cervical and ovarian cancer showed significant differences in the efficacy, indicating that chronotherapy can reduce the toxicity ratio of cisplatin treatment and enhance the efficacy (269). In addition, other chemotherapeutic agents showed optimal timing of administration to improve outcomes in bladder, colorectal, endometrial, and renal cancers (270–273). These studies found that the activity of several anticancer drugs may be limited by their side effects and toxicity on healthy cells, thus proving that temporal therapy offers the potential to optimize drug dosage and treatment duration to effectively eliminate cancer cells and reduce adverse effects, thereby preventing early drug resistance. Therefore, chronotherapy aims to optimize drug administration time to improve drug efficacy and safety without increasing drug dosage or changing drug type, maximize the antitumor effect of cancer chemotherapy by minimizing toxicity and adverse side effects, and increase tolerance to improve survival of patients.

## 7 Conclusion and perspectives

The data reviewed here demonstrate an overlap between the circadian clock and ***de novo*** FA synthesis in tumor lipid metabolism. Disorders of circadian clock can affect ***de novo*** FA synthesis, while dysregulation of this process can affect tumor development. The interaction between circadian clock and lipid metabolism plays a crucial role in tumor occurrence and progression, representing a potential mechanism influencing tumor development. However, several questions remain unanswered regarding the role of lipid metabolism in carcinogenesis, including the possible beneficial or harmful effects on cancer development that hinder the development of new therapeutic strategies. Our review establishes a connection between a disrupted circadian clock and key regulators of dysregulated lipid metabolism, as well as the molecular mechanisms underlying their effects on cancer. Nevertheless, to date, research has primarily focused on how the circadian clock regulates transcription factors involved in novo FA synthesis without thoroughly investigating key pathways through which both systems jointly influence tumor development. Although current studies have shown a strong association between circadian disruption, dysregulation of lipid metabolism, and cancer progression, there is still no systematic establishment of the mechanisms linking these factors together; thus future clinical and research efforts are necessary in this area. Future hypothesis-oriented studies should concentrate on specific interactions between circadian clock genes and lipid metabolism mechanisms to fully realize the clinical potential of their link in tumors.

To gain a deeper comprehension of how specific cancer genes obliterate tumor cells, targeting lipid metabolism and integrating it with the biological clock may offer a potent tool for optimizing cancer treatment. This approach also aims to enhance patients’ quality of life and survival rates by individualizing treatment time.

## Author contributions

ML: Writing – original draft. ZZ: Writing – original draft. YC: Software, Writing – original draft. TF: Methodology, Writing – original draft. QZ: Writing – review & editing. XT: Writing – review & editing.

## Glossary

**Table:**

FA	fatty acid
SREBP	sterol regulatory element binding protein
ACLY	ATP citrate lyase
ACC	acetyl-CoA carboxylase
FASN	fatty acid synthetase
SCD1	stearoyl-CoA desaturase 1
	
DEC	differential embryo-chondrocyte expressed gene
SCN	suprachiasmatic nucleus
TTFL	transcription-translation feedback loop
PER	Period Circadian Regulator
CRY	Cryptochrome
RORE	retinoic acid-related orphan receptor response elements
	
bHLH	basic helix-loop-helix
TAG	triacylglycerols
CPT1	carnitine palmitoyl transferase 1
FAO	fatty acid β-oxidation
TCA	tricarboxylic acid
ATP	adenosine triphosphate
MUFA	monounsaturated fatty acids
SCAP	SREBP cleavage-activating protein
ER	endoplasmic reticulum
COP II	coat protein complex—II
S1P	site 1 protease
S2P	Site 2 protease
PPAR α	Peroxisome proliferator-activated receptor alpha
PGJ2	prostaglandin J2
ACO	acyl-CoA oxidase
MLYCD	malonyl-CoA decarboxylase
PPRE	PPAR response element
PGC-1β	peroxisome proliferator-activated receptor γ
	
SIRT1	Sirtuin 1
NAD+	nicotinamide adenine dinucleotide
HDAC	nicotinamide adenine dinucleotide-dependent histone deacetylase
NAMPT	nicotinamide phosphoribosyltransferase
THCA	thyroid cancer
ccRCC	clear cell renal cell carcinoma
HCC	hepatocellular carcinoma
ESCC	esophageal squamous cell carcinoma
CRC	colorectal cancer
EC	endometrial cancer
BRCA	breast cancer
PAAD	pancreatic cancer
CSCs	cancer stem cells
NSCLC	non–small-cell lung cancer
OV	Ovarian cancer
NPC	nasopharyngeal carcinoma
SFA	saturated fatty acids
PC	pyruvate carboxylase
PI3K	phosphatidylinositol 3-kinase
RCC	renal cell carcinoma
CCGS	clock control genes
TCGA	Cancer Genome Atlas
MMP9	matrix metalloproteinase 9
GSC	glioblastoma stem cells.
